# Exploring pocket-aware inhibitors of BTK kinase by generative deep learning, molecular docking, and molecular dynamics simulations

**DOI:** 10.1039/d5ra04840k

**Published:** 2025-09-25

**Authors:** Li-Ting Zheng, Kun Qian, Jun Zhang, Meng-Ting Liu, Yi Li, Li-Quan Yang

**Affiliations:** a College of Agriculture and Biological Science, Dali University Dali China ylqbioinfo@gmail.com; b Key Laboratory of Bioinformatics and Computational Biology, Department of Education of Yunnan Province, Dali University Dali China; c Co-Innovation Center for Cangshan Mountain and Erhai Lake Integrated Protection and Green Development of Yunnan Province, Dali University Dali China; d College of Basic Medicine, Dali University Dali China; e College of Mathematics and Computer Science, Dali University Dali China yili@dali.edu.cn

## Abstract

Kinases play key roles in complex life processes such as signal transduction and cell cycle regulation. The dynamic conformation of kinase pockets, which is a critical target for the development of highly selective inhibitors, still presents significant challenges in terms of target selectivity, affinity, and off-target toxicity. In this study, we propose a computational framework to explore pocket-aware inhibitors targeting the J pocket of BTK kinase by integrating generative deep learning, molecular docking, and molecular dynamics simulations. We selected 5 candidate molecules by multi-step computational screening, which consisted of molecular clustering, molecular docking, and druggable evaluation, from 10 000 molecules generated through pocket-aware design. Molecular dynamics simulations showed that these 5 candidate compounds exhibited stable conformational dynamics, localized inhibitory effects, and favorable binding free energies during interaction with the BTK kinase. However, compared to the reference inhibitor (CFPZ), two candidates (C137 and C5598) demonstrated higher binding affinity and greater potential inhibitory activity. Further energy decomposition analysis revealed that Lys29 and Arg31 form key anchor points through electrostatic complementarity and salt bridge interactions, while Trp30 and Tyr70 enhance the stability of the complex through hydrophobic and aromatic stacking interactions, collectively establishing the molecular basis for efficient binding. Our study elucidates the binding mechanism of BTK kinase inhibitors, providing theoretical support for pocket-aware design of high-affinity, low-toxicity compounds. It highlights the novelty and target specificity of the candidate inhibitors and expands the applicability of deep learning in drug development for complex targets.

## Abstract 2

We propose a computational framework to explore pocket-aware inhibitors targeting the J pocket of BTK kinase by integrating generative deep learning, molecular docking, and molecular dynamics simulations.

## Graphical abstract

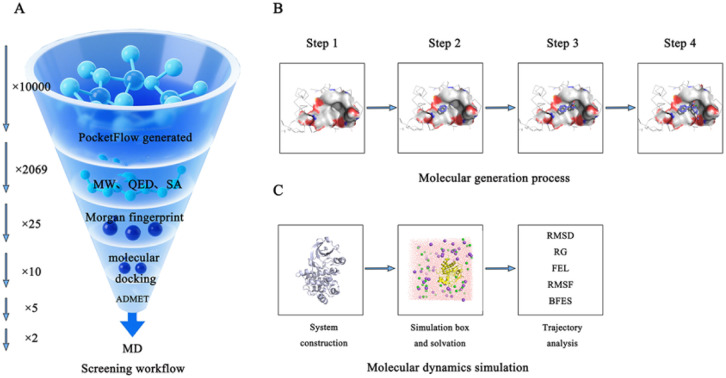

## 1. Introduction

Kinases are members of the phosphotransferase superfamily, one of the largest families of enzymes, accounting for approximately 2% of the human genome.^1^ Phosphotransferases catalyze the transfer of phosphate groups between molecules widely involved in signal transduction, metabolic regulation, and disease progression. As key members, kinases specifically phosphorylate target proteins, regulating essential biological processes such as cell division, signal transmission, and growth.^2^ This kinase-mediated phosphorylation mechanism is not only crucial for maintaining normal physiological functions but is also closely associated with various diseases, including cancer, autoimmune disorders, inflammatory diseases, cardiovascular conditions, and neurodegenerative disorders.^3^ Therefore, kinases have become important targets in modern medicine and drug development.^4^ Bruton's tyrosine kinase (BTK) is one of the most representative therapeutic targets. Since its identification in 1993 as the disease-causing gene responsible for X-linked agammaglobulinemia,^5,6^ its critical role in B cell development and functional regulation has become increasingly well researched. BTK is a central mediator in B cell receptor signaling, essential for B cell survival and differentiation. Its constitutive activation is a hallmark of several B cell malignancies, making BTK a validated target for therapeutic intervention.^7^ Structural and mechanistic insights into BTK have enabled the development of clinically approved small-molecule inhibitors, with expanding applications in autoimmune and inflammatory disorders.^8^ These advances underscore the importance of BTK as a druggable target and highlight the value of structural modeling in guiding next-generation inhibitor design.

As a prominent class of kinase-targeted therapeutics, kinase inhibitors have achieved significant clinical success. However, they still face critical challenges such as drug resistance and off-target toxicity, highlighting the urgent need for novel inhibitory strategies to enhance efficacy and safety. The development of kinase inhibitors has become an important direction in drug discovery and development since the first protein kinase inhibitor, imatinib, was approved for clinical use in 2001.^9–11^ As a major breakthrough in the targeted treatment of B-cell malignancies and autoimmune diseases, kinase inhibitors have made remarkable clinical progress in recent years. Representative agents such as ibrutinib, acalabrutinib, and zanubrutinib covalently bind to the ATP-binding site of kinases, irreversibly inhibiting its kinase activity and thereby blocking aberrant activation of the B-cell receptor signaling pathway. Clinical studies have shown that ibrutinib monotherapy achieved an overall response rate of up to 89% in a phase III trial for relapsed/refractory chronic lymphocytic leukemia, with median progression-free survival extended to 44 months.^12^ Second-generation inhibitors such as zanubrutinib demonstrated superior efficacy and safety compared to traditional regimens in the trial for the treatment of Wahl's macroglobulinemia by optimising kinase selectivity^13^ marking the era of precision in kinases-targeted therapy. However, the clinical application of this class of drugs still faces a number of challenges that need to be addressed. Firstly, the issue of drug resistance limits long-term efficacy, with approximately 30–50% of chronic lymphocytic leukemia patients developing acquired resistance during treatment, primarily due to genetic mutations.^14^ Mutations disrupt the covalent binding interface between the inhibitor and the kinase, resulting in a significant decrease in drug affinity. Secondly, off-target toxicity remains a key factor limiting drug tolerance. First-generation inhibitors, due to their lack of selectivity, often cause unexpected adverse reactions.^15^ These toxicities not only affect patients' quality of life, but also challenge the sustainability of the treatment regimen.

**Fig. 1:**
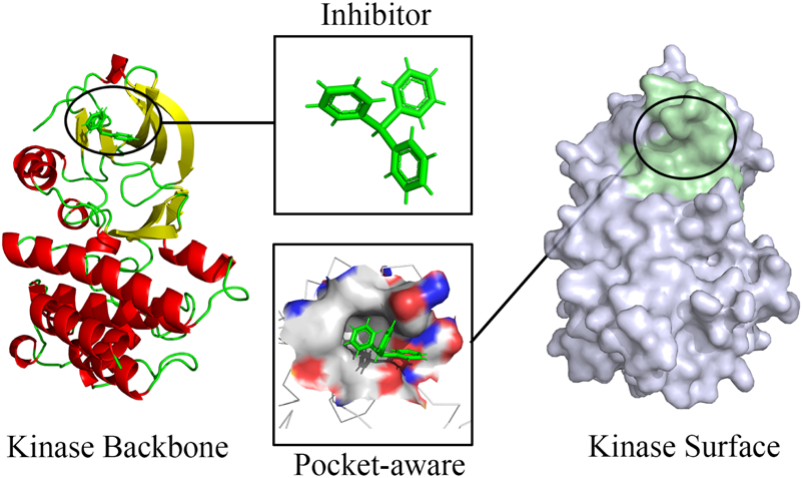
Schematic representation of the BTK kinase pocket and its inhibitor. The left panel shows the backbone structure of the kinase (PDB ID: 6BKW) together with the inhibitor CFPZ. The right panel illustrates the surface representation of the 6BKW kinase structure with the corresponding pocket highlighted. The red and blue regions on the pocket indicate negatively and positively charged areas, respectively, which are typically associated with hydrophilic interactions.

Currently, traditional ATP inhibitors target the highly conserved ATP-binding site, which is prone to mutations that lead to drug resistance. In contrast, the structurally diverse and less conserved J pocket has a lower mutation rate and is emerging as a promising target for developing next-generation inhibitors with high selectivity and low molecular weight. Although recent structural studies on AURKA first reported a hydrophobic pocket in the J-loop region that can be exploited by small molecules (the so-called Y-/J-pocket), and resolved several crystal structures of small-molecule complexes,^16^ similar structural sites had already been identified in other kinase families. A typical example is the PIF-binding pocket in PDK1 and related AGC kinases,^17,18^ which serves as a docking site for the hydrophobic motifs of downstream substrates and has been systematically characterized in structural and small-molecule regulation studies.^19–22^ Therefore, the J-pocket of AURKA is more appropriately regarded as a specific case or variant of a cross-family *N*-lobe hydrophobic site (PIF/Y-type), rather than a structural feature unique to AURKA. Based on this understanding, subsequent studies have revealed that the catalytic domain of BTK also harbors a similar J-pocket conformation, as illustrated in Fig. 1. Through systematic structural biology analysis, Oliver Laufkötter *et al.*^23^ elucidated the spatial localization of the J pocket in BTK, identifying it on the posterior side of the catalytic domain, oriented opposite to the ATP-binding site. Inhibitors form a stable thioether covalent bond with BTK Cys481 through a sulfur–Michael addition, which is accompanied by local conformational rearrangements around the active site. Multi-omics and computational studies have demonstrated that inhibitor occupancy and covalent modification can modulate the in/out equilibrium of the αC-helix and the conserved Lys–Glu salt bridge *via* an allosteric network, thereby biasing the kinase conformation toward an inactive state. This provides structural and dynamic insights for the development of allosteric or novel binding-mode inhibitors.^24,25^ The unique structural features of the J pocket distinguish it from conventional ATP-binding sites.^23,26^ Its shallow and flexible architecture can induce distinctive conformational changes upon ligand binding—for instance, J pocket occupation in AURKA has been shown to trigger αC-helix displacement, thereby modulating the active state of the catalytic center.^23^ Notably, J pockets exhibit high selectivity in their distribution across the kinase family (currently identified in only a few kinases such as AURKA and BTK), providing a structural basis for developing highly selective inhibitors.

However, the development of J pocket inhibitors still faces significant challenges. Current design strategies largely rely on static crystal structures, yet the J pocket undergoes allosteric modulation involving dynamic flipping of the thioether covalent bond at the Cys481 residue. This conformational flexibility leads to a considerable mismatch between the inhibitor binding modes and the actual conformational states, resulting in insufficient target residence time.^27–29^ Existing computational approaches also fall short in accurately capturing the entropy-driven contributions to binding free energy, leading to affinity prediction errors. For example, the binding strength of the second-generation inhibitor zanubrutinib was overestimated using Molecular Mechanics/Generalized Born Surface Area calculations.^30^ Moreover, high-throughput screening methods often lack sufficient coverage of the allosteric sites within the J pocket, necessitating time-consuming iterative optimization. Although earlier studies have explored the binding pathways of inhibitors to the BTK J pocket *via* conventional molecular dynamics simulations, parameterization biases have resulted in significant discrepancies in the predicted binding modes.^29^

With the widespread application of deep learning in drug molecule design, these challenges are gradually being addressed. Deep learning models can integrate multidimensional structural data to accurately capture the dynamic conformational changes of the BTK kinase J pocket, enabling the construction of high-precision models for predicting drug–pocket binding modes.^31^ This, in turn, facilitates the targeted optimization of inhibitor molecular compatibility and binding affinity.^32^ Specifically, deep reinforcement learning algorithms establish strategic exploration pathways within chemical space,^33^ allowing for the precise perception and generation of molecular structures that form stable interactions with key residues in the J pocket. This dynamic conformation-based recognition mechanism effectively reduces the off-target risk of candidate molecules and enhances the selectivity of drug design. Notably, deep learning models possess data-driven, self-adaptive parameter optimization capabilities, allowing them to integrate experimental data to dynamically refine molecular simulation parameters.^34^ This enables precise modeling of the J pocket microenvironment under complex biological systems, significantly improving the reliability of binding affinity predictions. At the technical implementation level, the synergistic application of deep generative models and reinforcement learning algorithms has established a closed-loop design system of “data integration – conformation prediction – molecule optimization”. This system mines hidden patterns within vast structural datasets to generate molecular candidates with specific interaction features, and iteratively optimizes the molecule–target interaction network through reinforcement learning strategies, ultimately yielding candidates that form highly stable interactions with key sites in the J pocket. This innovative design paradigm overcomes the limitations of traditional drug design, which often relies on empirical rules and limited structural data, and provides a systematic solution for the development of highly selective and high-affinity BTK inhibitors, paving the way for a more efficient and precise next-generation drug discovery process.^35^

In this study, we propose a computational framework that integrates generative deep learning, molecular docking, and molecular dynamics simulations to explore pocket-aware inhibitors targeting the kinase J pocket. The research focuses on uncovering the key interaction patterns between the J pocket and inhibitors, and evaluating how different chemical scaffolds influence pocket binding affinity. This direction not only expands our understanding of BTK inhibitor mechanisms but also offers an innovative strategy for designing highly selective, low-toxicity, and high-affinity targeted drugs. By accurately modeling the structural complexity and dynamic conformational changes of the J pocket, this study addresses core challenges in allosteric pocket recognition and binding prediction faced by traditional methods, significantly enhancing the reliability and efficiency of rational drug design. Our framework holds great potential to drive breakthroughs in therapeutic strategies for B-cell malignancies and autoimmune diseases, while extending the application boundaries of deep learning in the development of next-generation precision medicines for complex drug targets.

## 2. Materials and methods

### 2.1. Data collection and analysis

The co-crystal structure of BTK in complex with ligand CFPZ (PDB ID: 6BKW)^36^ was retrieved from the Protein Data Bank (PDB; https://www.rcsb.org/) and used as the reference structure for this study. This crystal structure was resolved at a resolution of 1.50 Å, with an *R*-factor of 0.164 and a free *R*-factor of 0.190, indicating high accuracy and reliability in atomic coordinates. To address the issue of local structural deficiencies in 6BKW, a homology modeling-based structure completion strategy was employed. After rigorous template screening, the high-resolution X-ray crystal structure 4RX5 chain A was selected as the template. Based on this template, an initial model was constructed and subsequently refined to generate a high-quality 3D structural model of BTK, providing a solid and reliable structural foundation for further investigation. Heteroatoms and water molecules were removed using PyMOL software,^37^ while retaining the complete BTK kinase catalytic domain. This preprocessing step effectively eliminated nonspecific interactions that might interfere with subsequent calculations, ensuring the structural purity and accuracy of the docking system.

### 2.2. Overview of the generation, docking, screening, and simulation workflow

**Fig. 2:**
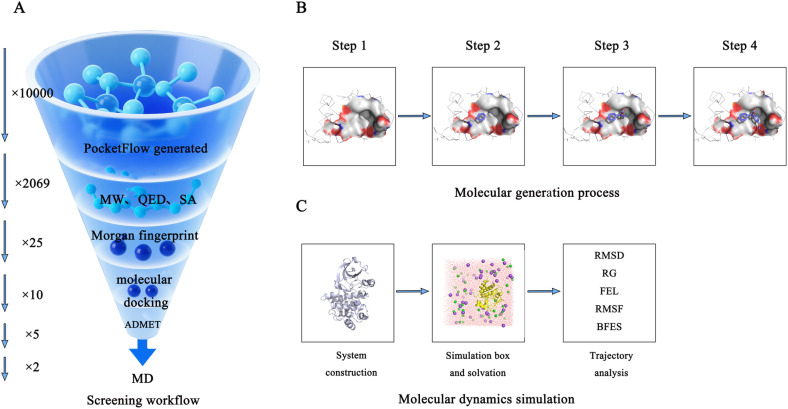
Workflow diagram. (A) Overall workflow of molecular generation and screening. (B) Process of molecular generation using our deep learning model. (C) Molecular dynamics simulation procedure and subsequent trajectory analysis.

Based on the dynamic conformational characteristics of the BTK J pocket, an AI-driven generative deep learning algorithm was employed to perform virtual screening, enabling systematic perception of the J pocket chemical space and generation of novel ligand molecules. As shown in Fig. 2, firstly, based on the crystal structure of the J-pocket, and incorporating conformational diversity information reported in the literature,^38,39^ the algorithm generated 10 000 candidate molecules. These were then subjected to a multidimensional filtering strategy to obtain a subset of molecules with favorable drug-like potential. Secondly, clustering algorithms were used to divide the filtered candidates into a limited number of feature-based clusters according to their morphological and chemical properties, thereby ensuring structural diversity. A representative molecule was selected from the center of each cluster, and molecular docking was performed using a known active compound as a reference to identify candidates with high binding affinity. Furthermore, pharmacokinetic profiles, metabolic stability, and toxicity risks of the shortlisted molecules were comprehensively assessed to determine the most promising lead compounds. Finally, to verify the binding stability, multiple-replica molecular dynamics simulations were performed using simulation software. By analyzing the root–mean–square deviation, radius of gyration, root–mean–square fluctuation of residues, and binding free energy of the protein-ligand complexes, the stable binding mode of the candidate molecules to the J pocket was confirmed. Our study establishes a systematic design framework for J pocket inhibitors through the deep integration of artificial intelligence algorithms and multiscale computational techniques, offering an innovative paradigm for the development of highly selective and stable BTK inhibitors.

### 2.3. Pocket-aware candidate design and analysis

We employed the PocketFlow model^40^ to generate novel ligand molecules within the protein binding pocket. The generation process involves several key steps.

(1) Input of environmental information: the binding pocket (derived from the target protein) and the partially generated molecular fragments are used as environmental inputs.

(2) Context encoder: a multi-head equivariant graph attention network composed of stacked interaction blocks (including message-passing and attention modules) encodes the environment to extract contextual features *C*^(*t*−1)^.

(3) Focal net: based on *C*^(*t*−1)^, the model selects a focal atom from the existing fragment to serve as the origin of a local coordinate system. Saturated atoms are excluded, and for the first generated atom, a focal site is chosen from the pocket wall.

(4) Atom flow module for generating new atom types: the atom flow module converts the hidden features of the focal atom (*v*_*t*−1_^f^,*v*_*t*−1_^f^), and a latent variable *z* randomly sampled from a standard normal distribution *N* (0, 1) into a probability distribution *P*(*a*^(*t*)^|*C*^(*t*−1)^) over possible atom types, thereby determining the type of the new atom.

(5) Position predictor module for calculating atom positions: taking the focal atom as the origin of a local coordinate system, the position predictor module uses the features of the focal atom and the embedding of the new atom type as inputs to compute the relative position Δ*r*^(*t*)^, thereby obtaining the coordinates *r*^(*t*)^ of the new atom. This module is implemented as a Mixture Density Network (MDN) built upon a Geometric Double Bottleneck Perceptron (GDBP), enabling the direct generation of the *xyz* coordinates of the new atom. To avoid unrealistic bond lengths, the distance between the new atom and the focal atom is restricted to within 2 Å.

(6) Bond flow module generates covalent bonds: to avoid unrealistic bond lengths in the generated molecules, the bond flow module only considers atoms within 4 Å of the focus atom as candidates for forming covalent bonds with the newly generated atom. The module samples *z* from a normal distribution and computes the bond type distribution *P*(*e*^(*t*)^|*C*^(*t*−1)^,*a*^(*t*)^, *r*^(*t*)^) through the feedforward transformation of the normalizing flow. In this process, a trihedral equivariant attention module is introduced to capture geometric constraints, and chemical valence constraints are explicitly applied to check whether the current covalent bonds exceed the allowable valence of each atom. If a newly generated covalent bond exceeds an atom's maximum valence, or forms alert structures such as O–O, O–N, C=C=C, or three-membered rings, the bond is removed and a different bond is resampled.

(7) Adding new components and determining termination: the newly generated atom type *a*^(*t*)^ coordinates *r*^(*t*)^ and covalent bonds *e*^(*t*)^ are added to the environment as input for the next generation step. The generation process terminates under any of the following conditions: no atom can be predicted as the focus atom; the number of generated atoms reaches a predefined maximum; or the number of resampling attempts reaches a predefined limit. Resulting in the output of small molecules with high drug-likeness and chemically reasonable structures.

Using the RDKit cheminformatics toolkit in combination with Python programming,^41^ we systematically calculated key physicochemical properties for both the 10 000 AI-generated molecules and the original seed compounds. These properties included molecular weight (MW), synthetic accessibility score (SA), and quantitative estimate of drug-likeness (QED). The SA score is calculated based on molecular complexity, the types of functional groups, and synthetic feasibility derived from known molecules, while the QED score incorporates Lipinski's Rule of Five and other molecular properties such as molecular weight, polar surface area, the number of hydrogen bond donors/acceptors, and lipophilicity. In this study, an initial systematic screening was performed on 10 000 small molecules. Based on criteria rooted in medicinal chemistry rationality, a total of 2069 candidate molecules were selected. These molecules were then structurally characterized using the Morgan fingerprint method, which captures topological features, and classified into 25 distinct clusters through the K-means clustering algorithm,^42^ according to their morphological and chemical properties. To evaluate the binding potential of candidate molecules with the target protein, a representative molecule was selected from the center of each cluster for molecular docking experiments, ensuring structural diversity among the compounds.

### 2.4. Molecular docking

AutoDock Vina 1.2.0 (ref. 43) was utilized as the primary tool for molecular docking. The 25 small molecules selected from the cluster centers based on Morgan fingerprints were converted into pdbqt format. These ligands were then docked to the BTK target protein, which had been constructed *via* homology modeling. Docking results were evaluated based on binding scores and ranked accordingly. By comparing these scores with that of the reference crystal complex, the top 10 compounds exhibiting the most favorable binding free energies were identified as potential lead candidates for further study.

The key parameters for molecular docking were configured as follows, first, the crystal structure file of the BTK protein complexed with the small-molecule inhibitor, BTK/CFPZ.pdb, was loaded into AutoDock Vina, along with its co-crystallized ligand CFPZ.pdb, to accurately define the binding site. The geometric center of the binding pocket was then determined by precise measurement within the software, yielding coordinates of 44.983, −0.764, 8.445. Next, the spatial extent of the binding site was evaluated to ensure full coverage of the active region by the docking box, which was ultimately set to a size of 40 Å × 40 Å × 40 Å. These parameters were incorporated into the AutoDock Vina configuration file to define the docking grid. A grid spacing of 0.375 Å was used, and ten ligand conformations were generated per docking run to improve sampling diversity. Docking calculations were carried out using the Lamarckian Genetic Algorithm with default parameters. The conformation with the lowest binding energy was selected as the optimal protein–inhibitor complex structure for subsequent molecular dynamics simulations. This systematic docking workflow provides a robust structural foundation for downstream drug design and optimization efforts.

### 2.5. Drug-likeness evaluation

The ADMET (Absorption, Distribution, Metabolism, Excretion, and Toxicity) profiles of the ten candidate compounds were systematically evaluated using the online platform ADMETlab 3.0 (https://admetmesh.scbdd.com/).^44–46^ Specifically, key pharmacokinetic parameters were calculated, including blood–brain barrier (BBB) permeability, human intestinal absorption (HIA), Caco-2 cell permeability, and MDCK cell permeability. In addition to pharmacokinetics, comprehensive toxicity assessments were conducted. These included predictions of hERG channel inhibition potential, AMES mutagenicity, carcinogenicity, acute oral toxicity, and human hepatotoxicity. Based on ADMETlab 3.0 integrated scoring system and toxicity indices, the top five compounds were selected, demonstrating optimal profiles in both pharmacokinetic behavior and toxicity risk. These selected molecules represent promising lead candidates, providing a strong theoretical and experimental foundation for early-stage drug discovery. This systematic ADMET evaluation strategy lays a solid groundwork for further drug optimization and development.

### 2.6. All-atom molecular dynamics simulations

All molecular dynamics (MD) simulations were performed using the GROMACS 2021 software suite,^47^ in conjunction with the AMBER99SB-ILDN force field.^48^ Simulations were conducted on the experimentally determined BTK/CFPZ crystal structure, representing a prototypical complex of the J pocket, as well as on five modeled complexes formed by the binding of *de novo* generated ligands to their corresponding receptors. Each complex was solvated in a dodecahedral box filled with TIP3P water molecules,^49^ ensuring a minimum distance of 1.2 nm between any solute atom and the box edge. Periodic boundary conditions were applied in all directions. Counterions were added to neutralize the net charge of each system, and the ionic strength was adjusted to a final salt concentration of 150 mM. Initial energy minimization was carried out using the steepest descent algorithm until convergence was achieved, *i.e.*, when no significant energy fluctuations were observed. Prior to production runs, each system underwent a two-phase equilibration, a 500 ps NVT (constant number of particles, volume, and temperature) phase followed by a 300 ps NPT (constant number of particles, pressure, and temperature) phase. These steps were designed to stabilize the system and provide a reliable starting configuration for subsequent long-term simulations.

To improve the robustness and statistical reliability of the simulations,^50^ three independent 100 ns production MD runs were carried out for each complex, with initial velocities assigned according to a Maxwell–Boltzmann distribution at 310 K. In the production simulations, a 2 fs integration time step was used. All covalent bonds were constrained using the LINCS algorithm.^51^ Long-range electrostatics were calculated using the particle-mesh Ewald (PME) method,^52^ with a real-space cutoff of 1.2 nm, a Fourier grid spacing of 0.12 nm, and a fourth-order interpolation scheme. For van der Waals (vdW) interactions, a twin-range cutoff was applied, with neighbor lists and vdW interactions truncated at 1.0 nm and 1.2 nm, respectively. Temperature was maintained at 300 K using a velocity-rescaling thermostat^53^ with a coupling constant of 0.1 ps, applied separately to solute and solvent. System pressure was maintained at 1 bar using the Parrinello–Rahman barostat^54^ with a coupling constant of 2.0 ps. Coordinates were saved every 2 ps for subsequent trajectory analysis.

### 2.7. Trajectory analysis

To comprehensively investigate the structural and dynamic features of the protein–ligand complexes, we employed MD simulations in combination with a suite of analytical tools. First, the root mean square deviation (RMSD) of the protein Cα atoms and ligand heavy atoms relative to their initial coordinates was calculated using the GROMACS tool ‘gmx rms’. This analysis provided insight into the global conformational stability of the complexes throughout the simulation. Next, the radius of gyration (*R*_g_) of the protein was computed using ‘gmx gyrate’ to evaluate changes in global compactness and structural organization during the simulation. To examine local flexibility at the residue level, root mean square fluctuation (RMSF) values were calculated using ‘gmx rmsf’, providing a residue-specific profile of dynamic fluctuations over time. To further explore the conformational free energy landscape (FEL) of the complexes, FEL plots were constructed from the MD trajectories using custom Python scripts. In addition, per-residue free energy contributions were estimated to identify key residues involved in complex stabilization and functional interactions. Collectively, these analyses offer a comprehensive view of the molecular interactions and dynamic behavior underlying protein–ligand binding.

### 2.8. Binding free energy calculations

The molecular mechanics Poisson–Boltzmann surface area (MM-PBSA) method^55^ is one of the most widely used approximate approaches for calculating binding free energies (BFEs). In this study, we systematically evaluated the binding free energies of each protein–ligand complex using the MM-PBSA method implemented in the ‘gmx_mmpbsa’ module within the GROMACS software package. Specifically, the ‘gmx_mmpbsa’ tool analyzes the MD simulation trajectories to compute the binding free energy associated with each sampled frame. The binding free energy (Δ*G*_bind) of the protein–ligand complex was calculated using the following equation:Δ*G*_bind_ = *G*_complex_ − *G*_ligand_ − *G*_protein_where *G*_complex_ represents the total free energy of the protein–ligand complex, *G*_protein_ denotes the free energy of the isolated receptor, and *G*_ligand_ corresponds to the free energy of the isolated ligand. This approach enables a quantitative evaluation of the stability of protein–ligand interactions, providing crucial energetic insights for subsequent drug design and optimization. Moreover, the MM-PBSA-based strategy enhances the predictive accuracy of binding free energies and offers theoretical support for elucidating the molecular mechanisms underlying biomolecular interactions.

## 3. Results

### 3.1. Description and evaluation of candidate molecules

To systematically evaluate the distributional differences between candidate molecules and the reference compound within chemical space, we conducted a comprehensive statistical analysis on 10 000 candidate molecules and their reference counterpart. Key molecular properties analyzed included MW, SA score, and QED score—parameters widely recognized for their relevance in drug design and compound prioritization. The SA score, ranging from 1 (easy to synthesize) to 10 (difficult to synthesize), quantifies the synthetic feasibility of a compound, whereas the QED score, ranging from 0 to 1, reflects drug-likeness, with higher values indicating more favorable drug-like characteristics.

**Fig. 3:**
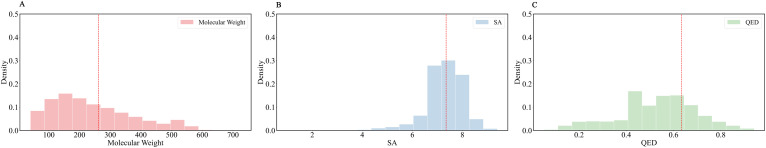
Distribution of molecular properties for the 10 000 candidate compounds. (A) Distribution of molecular weight (MW), (B) distribution of synthetic accessibility (SA), and (C) distribution of drug-likeness (QED) scores for the candidate compounds. The red dashed lines represent the reference values of compound CFPZ, which are used for comparative evaluation of the property distributions of the generated molecules relative to those of the known active compound.

As shown in Fig. 3, the MW of the candidate compounds were primarily distributed between 100 and 200 Da, whereas the reference compound CFPZ has a molecular weight of 262 Da. To ensure sufficient pharmacophoric complexity and binding potential, we selected compounds within the 200–400 Da range for further analysis. The SA and QED scores of CFPZ were 7.35 and 0.63, respectively. Based on these values, we applied a dual-filtering criterion—SA score < 8 and QED score ≥ 0.6—to identify compounds that are both synthetically accessible and possess favorable drug-likeness. A total of 2069 compounds met these criteria.

**Fig. 4:**
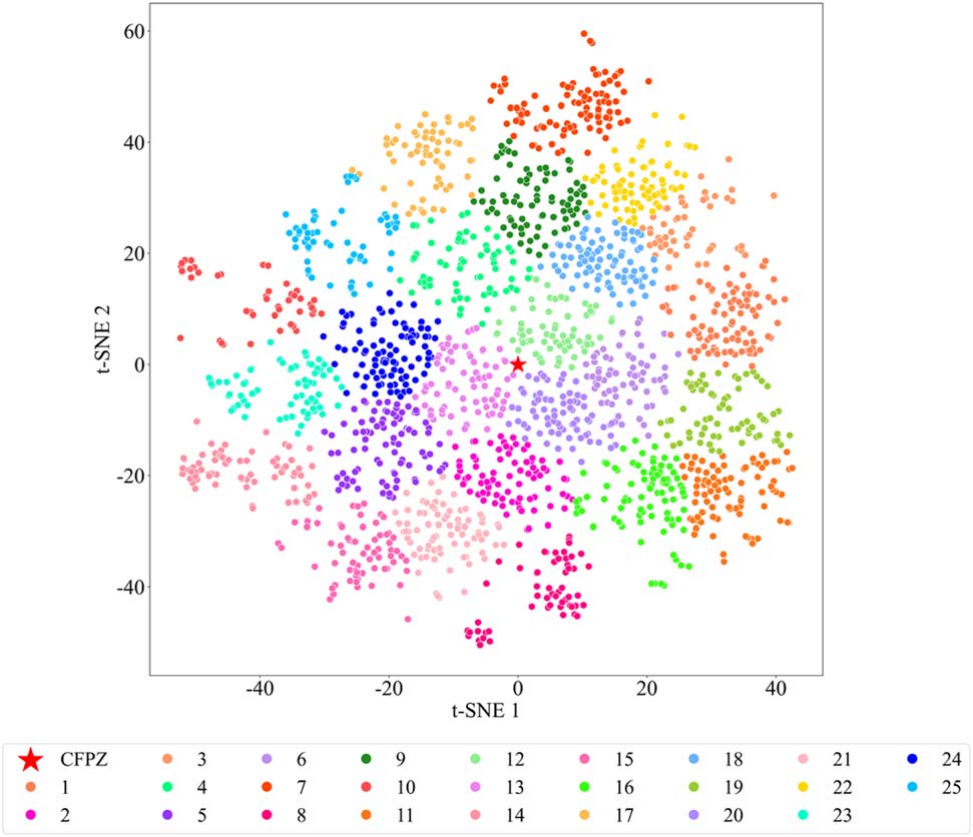
Morgan fingerprint map of the reference molecule and 2069 candidate compounds. Different colors represent different cluster labels, which distinguish the clustering results based on structural similarity, reflecting the molecular structural diversity and distribution characteristics. The red pentagram marks the reference molecule CFPZ, which serves as a reference for structural comparison.

Next, we performed clustering analysis on the filtered candidate molecules based on their molecular morphology and structural features. Each molecule was encoded using a 1024-dimensional Morgan fingerprint, which captures its topological characteristics. The molecules were then grouped into 25 distinct clusters based on fingerprint similarity. To visualize the high-dimensional molecular data, we applied *t*-distributed Stochastic Neighbor Embedding (*t*-SNE) to project the 1024-dimensional fingerprint space into a 2D plane. The resulting *t*-SNE 1 and *t*-SNE 2 axes represent the two reduced dimensions, preserving the relative similarity relationships among molecules. Points that are close together on the plot indicate high similarity in the original high-dimensional space, while those further apart represent more distinct molecular features. As shown in Fig. 4, each cluster is denoted by a unique color and numeric label. Molecules within the same cluster exhibit similar Morgan fingerprint patterns. Densely packed regions indicate areas where molecules share highly similar morphological and chemical features, whereas sparser regions suggest transitional areas between clusters, with molecules exhibiting mixed characteristics. The clear boundaries between many clusters further highlight the distinctiveness of the molecular subgroups. A red pentagram marks the reference compound CFPZ, serving as the control in this study. Notably, clusters 13, 19, and 20 are located in close proximity to CFPZ in the *t*-SNE space. Given that the *t*-SNE algorithm is designed to preserve local similarity structures during dimensionality reduction, the close placement of these clusters to the reference molecule implies a high degree of similarity in their encoded features. This observation suggests that clusters 13, 19, and 20 may share key structural or functional characteristics with the reference compound, potentially placing them in the same functional group or endowing them with similar biological properties. These findings offer important clues for further functional annotation and classification analysis.

**Fig. 5:**
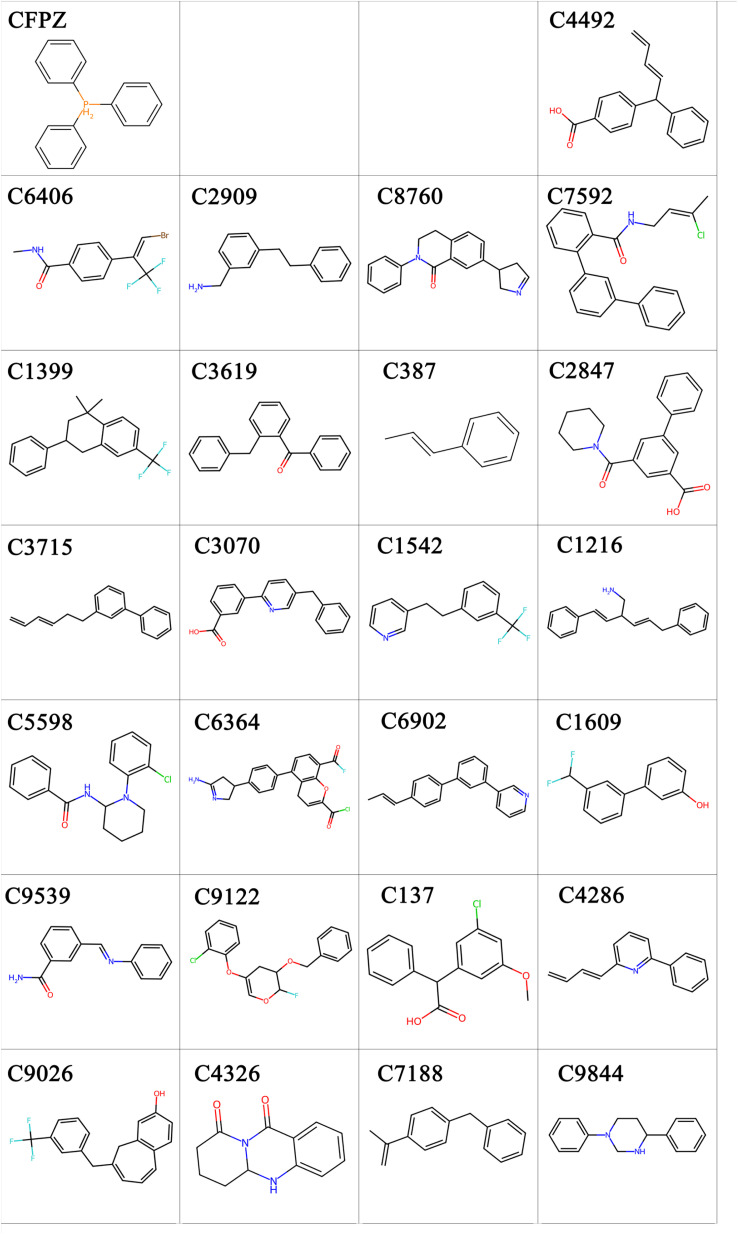
Representative core chemical structures of the 25 candidate compounds. Each compound is labeled with its corresponding cluster number, reflecting its classification within the structural fingerprint space. The figure also includes compound CFPZ, which serves as a reference structure for comparative analysis. Different colors are used to highlight various types of functional groups present in the compounds.

In the field of drug design, compounds featuring a benzene ring as the core scaffold are widely utilized due to their unique structural advantages. Based on the aforementioned analysis, one representative molecule was selected from the center of each cluster, resulting in a total of 25 compounds for further evaluation. The conjugated electron system of the benzene ring imparts aromaticity and hydrophobicity to the molecules, making it a crucial structural motif in medicinal chemistry. As shown in Fig. 5, all 25 compounds investigated in our study are built upon a benzene-based framework, with the introduction of diverse functional groups enabling precise modulation of molecular properties. Specifically, the introduction of hydroxyl groups, as seen in C137, C1609, and C4492, serves as hydrogen bond donors or acceptors, significantly enhancing molecular aqueous solubility. Halogen atoms, such as chlorine in C137 and fluorine in C1542, C1609, and C6406, adjust the molecule's lipophilicity and partition coefficient, thereby optimizing pharmacokinetic properties. Amino or imino groups, found in compounds like C1216, C2909, and C6406, act as hydrogen bonding sites, enabling specific interactions with biological targets and improving binding affinity. Carbonyl groups, as in C137, C4326, and C9539, are often present in esters, ketones, or amides, contributing to molecular polarity and participating in the modulation of biological activity. The presence of ether linkages, such as in C137, increases molecular flexibility and enhances membrane permeability. Amide bonds, as seen in C4326, C7592, and C9539, function as key pharmacophores that can form stable hydrogen-bonding networks with targets, reinforcing drug–target interactions. It is worth noting that the reference compound CFPZ, a triphenylmethane derivative, contains only the benzene ring scaffold. Although it exhibits the stability and hydrophobic character typical of aromatic systems, its lack of functional group diversity limits its drug-like versatility. In contrast, the other 25 candidate compounds enhance the benzene core with strategically introduced hydroxyl, halogen, amino, and other functional groups. This combinatorial functionalization strategy leads to systematic improvements in solubility, target affinity, and metabolic stability. Such diversified functional design based on a benzene ring scaffold not only exemplifies the core principle of structure–activity relationships in medicinal chemistry but also provides structural diversity to accommodate a wide range of biological targets. This approach lays a solid foundation for exploring SAR and supports multi-scenario applications in drug discovery and development.

### 3.2. Docking assessment

To enhance the efficiency and accuracy of drug screening, this study employed molecular docking as a central strategy for identifying promising small-molecule candidates. As a pivotal tool in structure-based drug design, molecular docking has also gained widespread application in ligand-based computer-aided drug discovery, particularly for target identification and lead compound selection. The fundamental principle of docking involves simulating the binding conformations and interaction energies between small-molecule ligands and protein targets, thereby predicting their potential binding modes and affinities. This enables high-throughput screening of large compound libraries. Compared to traditional experimental approaches, molecular docking significantly reduces both time and resource costs, while improving hit rates during the early stages of drug discovery. As such, it has become an indispensable component of modern, computationally driven drug development workflows.

We conducted an in-depth molecular docking analysis of the 25 candidate molecules selected in the previous step. Initially, we performed a preliminary screening based on affinity score (Table S1). The reference complex BTK/CFPZ exhibited an affinity score of −6.8 kcal mol^−1^. To identify candidate compounds with higher binding affinity, we selected complexes with affinity scores superior to that of the reference structure. As a result, the top 10 compounds with the best scores were identified, BTK complexed with compounds C137, C1216, C1399, C2847, C2909, C5598, C6902, C7592, C9026, and C9539. These complexes displayed affinity scores ranging from −7.0 to −8.6 kcal mol^−1^. Compared to the reference structure, all of these compounds demonstrated lower affinity energies, indicating more stable interactions with the target protein.

**Fig. 6:**
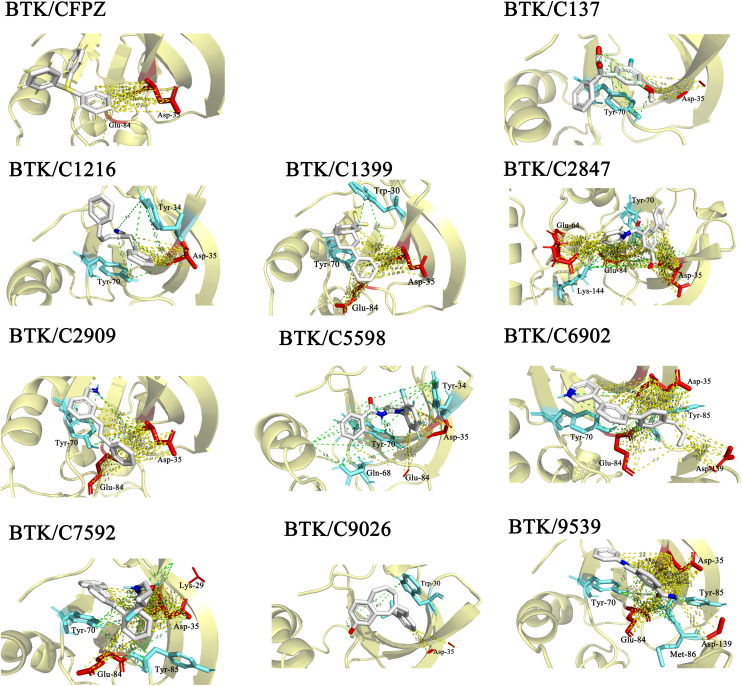
Molecular docking poses of the candidate inhibitors. The figure illustrates the 3D interactions between BTK and the 10 representative candidate inhibitors (C137, C1216, C1399, C2847, C2909, C5598, C6902, C7592, C9026, and C9539) identified through docking-based screening. The reference complex BTK/CFPZ is also included for comparison. Hydrogen bonds are represented by green dashed lines, with the interacting residues shown in cyan. Electrostatic interactions are represented by yellow dashed lines, with the corresponding residues displayed in red. Key residues within a 4 Å radius are highlighted to show potential binding interactions.

We then carried out a detailed intermolecular interaction analysis for these top 10 complexes (Table 1). Key amino acid residues such as Trp30, Asp35, Tyr70, Trp4, and Val36 were identified as critical contributors to the interactions between the small-molecule inhibitors and the apo form of BTK. All 10 complexes displayed strong hydrogen bonding interactions, along with notable hydrophobic contacts. As illustrated in Fig. 6, the docking conformations of the top 10 ligands in the BTK binding pocket are shown, highlighting the key residues involved in interactions within 4 Å of each ligand. These docking poses demonstrate that the candidate molecules are well-positioned within the BTK active site, forming stable interactions with essential amino acid residues. These results provide a solid structural foundation for evaluating the target-binding capabilities and potential activity of the generated compounds, and offer valuable insights for subsequent optimization and rational drug design.

**Table 1:** Molecular affinity scores and contact residues of BTK/CFPZ with the top 10 candidate molecules

	BTK/CFPZ	BTK/C 137	BTK/C 1216	BTK/C 1399	BTK/C 2847	BTK/C 2909	BTK/C 5598	BTK/C 6902	BTK/C 7592	BTK/C 9026	BTK/C 9539
Affinity score (kcal mol^−1^)	−6.8	−7.0	−7.3	−7.3	−8.5	−7.4	−7.4	−8.2	−7.2	−8.6	−7.1
Hydrogen bonds	Trp30, Tyr34, Asp35, Tyr70	Tyr70	Tyr34, Tyr70	Trp30, Asp35, Tyr70	Asp35, Tyr70	Asp35, Tyr70	Tyr34, Gln68, Tyr70	Tyr70, Tyr85	Asp35, Tyr70, Tyr85	Trp30	Tyr70, Tyr85, Met86
Hydrophobic interactions	Trp4, Trp30	Trp4, Trp30, Val36	Trp4, Ile6, Trp30, Val36	Trp4, Ile6, Trp30, Val36, Ile82	Trp4, Trp30, Val36	Trp4, Ile6, Trp30, Val36, Ile82	Trp4, Trp30, Val36	Trp4, Trp30, Val36	Trp4, Trp30, Val36	Trp4, Ile6, Trp30, Val36, Ile82	Trp4, Trp30, Val36, Met8

### 3.3. Drug-likeness evaluation

To enhance the success rate of candidate compounds entering clinical trials and reduce potential safety risks during early-stage drug development, systematic evaluation of ADMET properties has become a critical step in the drug design process. We performed a comprehensive ADMET prediction analysis on the 10 candidate compounds identified through molecular docking (Table S2). The analysis covered key pharmacokinetic and toxicity-related parameters, including oral bioavailability, blood–brain barrier permeability, potential mutagenicity, and hepatotoxicity. Based on a multidimensional assessment of these factors, 5 compounds exhibiting favorable pharmacokinetic profiles and low toxicity risks were ultimately selected as core candidates for further optimization and validation (Table 2).

**Table 2:** The ADMET (Absorption, Distribution, Metabolism, Excretion, and Toxicity) spectra of the top 5 candidate compounds and the reference compound CFPZ^a^

	Caco-2	HIA	BBB	MDCK	Pgp inhibitor	Pgp substrate	hERG blockers	AMEs muta genicity	Carcinogenicity	Acute toxicity rule	H-HT
CFPZ	−4.965	0.0	0.313	−4.706	0.139	0.999	0.0	0.0	1.0	0	0.999
C137	−4.866	0.004	0.01	−4.479	0.002	0.007	0.131	0.09	0.11	0	0.664
C2909	−4.963	0.137	0.486	−4.625	0.995	0.263	0.771	0.286	0.051	0	0.442
C5598	−4.641	0.0	0.979	−4.829	0.78	0.16	0.434	0.235	0.297	0	0.698
C2847	−4.687	0.004	0.014	−4.715	0.0	0.027	0.182	0.263	0.33	0	0.68
C1216	−5.048	0.002	0.939	−5.072	0.869	0.025	0.577	0.565	0.122	0	0.551

a The reference thresholds and predictive models for ADMET parameters were obtained from established computational resources, including Pires *et al.*, (2015), Cheng *et al.*, (2012), and have also been widely applied in recent computational drug discovery studies Hemavathi K N *et al.*, 2024.^56–58^

In the evaluation of pharmacokinetic properties, we systematically analyzed the intestinal absorption, membrane permeability, and drug metabolism characteristics. The HIA prediction showed that the HIA value for the reference CFPZ was 0.0, indicating no intestinal absorption capability. In contrast, the candidate compounds demonstrated significant advantages: C137 had an HIA value of 0.004, C2909 reached 0.137, C2847 was 0.004, and C1216 was 0.002. These positive HIA values confirm that the candidate compounds possess significantly better intestinal absorption capabilities than CFPZ, with greater potential for oral bioavailability, laying a solid foundation for clinical drug administration route selection. For the critical pharmacokinetic parameter of membrane permeability, the study focused on BBB permeability and MDCK permeability as core evaluation metrics. In terms of BBB permeability, the CFPZ had a BBB value of 0.313, while C5598 had a BBB value of 0.979, C2909 was 0.486, and C1216 reached 0.939. Notably, C5598 and C1216 exhibited significantly higher BBB permeability than CFPZ, suggesting that these compounds may have better potential for penetrating the blood–brain barrier and exerting targeted effects in the development of drugs for neurodegenerative diseases. For MDCK permeability, the CFPZ had a value of −4.706, while C137 was −4.479, C2909 was −4.625, and C2847 was −4.715. These data indicate that the candidate compounds exhibit superior transmembrane transport capacity, with significantly better membrane permeability than the reference CFPZ. In terms of drug metabolism characteristics, the evaluation of *P*-glycoprotein substrates and inhibitors showed that the *P*-glycoprotein substrate value for CFPZ was 0.999, indicating a high risk of efflux. Among the candidate compounds, C137 had a *P*-glycoprotein substrate value of only 0.007, C2847 was 0.027, and C1216 was 0.025, significantly reducing the efflux risk. Furthermore, the *P*-glycoprotein inhibitor values revealed that C137 had a value of 0.002, C2909 reached 0.995, and C1216 was 0.869, demonstrating better performance in modulating drug interactions compared to the reference compound.

In the context of toxicity risk assessment, our study established a comprehensive evaluation framework focusing on AMES toxicity, carcinogenicity, acute oral toxicity, and human hepatotoxicity. Regarding genotoxicity, the AMES mutagenicity value for the reference compound CFPZ was 0.0, while candidate compounds C137, C2909, and C2847 exhibited values of 0.09, 0.286, and 0.263, respectively. Comparative analysis indicates that the candidate compounds do not demonstrate higher mutagenic potential; in fact, some molecules exhibit stable profiles in terms of genotoxicity control. Carcinogenicity assessment further highlights the safety advantages of the candidate compounds, while the reference compound CFPZ has a carcinogenicity score of 1.0, the values for C137, C2909, and C1216 are significantly lower, at 0.11, 0.051, and 0.122, respectively. In addition, human hepatotoxicity evaluations show that the H-HT value for CFPZ is 0.999, indicating a high toxicity risk. In contrast, the candidate compounds C137, C2909, and C1216 have H-HT values of 0.664, 0.442, and 0.551, respectively—all substantially lower than that of CFPZ—suggesting a reduced risk of hepatotoxicity and a stronger safety profile overall.

### 3.4. Designed candidates strategically inhibit global dynamic of kinase

**Fig. 7:**
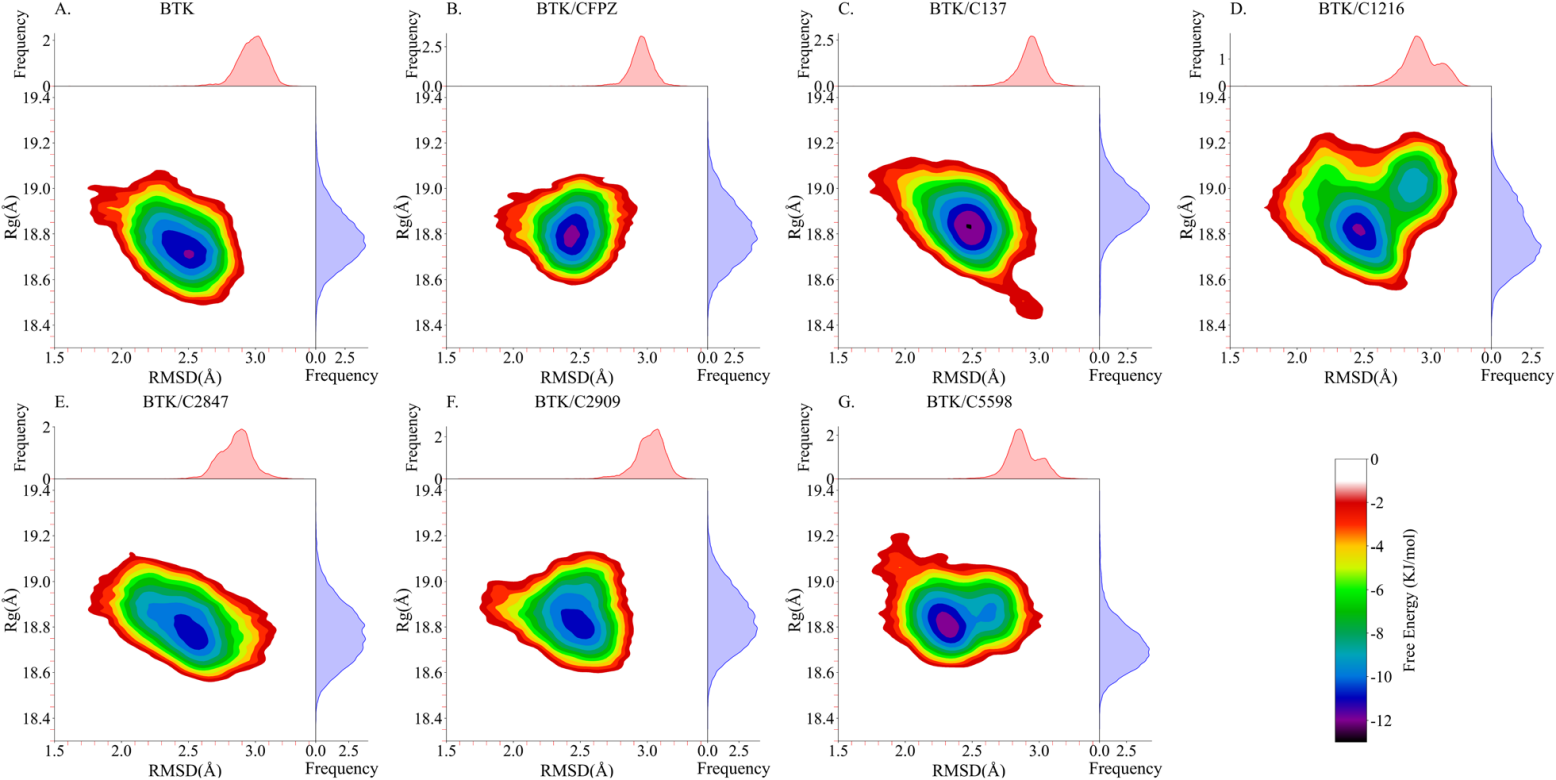
Free energy landscapes. (A) Free energy landscape of the apo BTK structure, (B) free energy landscape of the BTK/CFPZ complex, (C–G) free energy landscapes of BTK complexed with compounds C137, C1216, C2847, C2909, and C5598, respectively. Each figure displays Root Mean Square Deviation (RMSD, Å) on the *x*-axis, indicating structural deviation from the initial conformation, and radius of gyration (*R*_g_, Å) on the *y*-axis, reflecting molecular compactness. Color gradients represent different free energy distributions.

To further investigate the conformational stability changes of the protein–ligand complexes throughout the 100 ns MD simulations, we constructed FEL, as shown in Fig. 7. The FELs were generated based on three-dimensional data comprising the RMSD, Rg (Fig. S1 and S2), and Gibbs free energy of the protein structures. The resulting two-dimensional free energy landscapes are color-coded using a gradient scale from high (red) to low (purple) energy levels, visually representing the relative stability of different conformational states.

Analysis of the FELs revealed that the most stable conformations of all complexes correspond to the global minima in free energy. Fig. 7A and B illustrate the free energy distributions of the apo BTK structure and the BTK/CFPZ complex, respectively. In both cases, the high-frequency RMSD regions are concentrated within the 2.05–2.55 Å range, indicating relatively consistent structural fluctuations. Similarly, the *R*_g_ values are predominantly distributed between 18.65–18.95 Å. The minimum free energy in both structures reaches −12 kJ mol^−1^, suggesting a reasonable degree of conformational stability. Fig. 7C to G present the FELs of BTK in complex with compounds C137, C1216, C2847, C2909, and C5598, respectively. For these complexes, RMSD values range from 1.55–3.05 Å, with the densest conformational clusters centered around 2.05–2.65 Å. The *R*_g_ values span 18.55–19.15 Å, with high-frequency regions primarily between 18.75-18.95 Å. Notably, This complex displays a single main energy well, with the lowest free energy reaching −13 kJ mol^−1^—the lowest among all systems in a single well distribution—indicating excellent thermodynamic stability and strong binding. This makes BTK/C137 one of the most stable complexes in the analysis. In contrast, BTK/C2847 and BTK/C2909 (Fig. 7E–F) exhibit minimum free energy values around −10 kJ mol^−1^, which do not reach the stability levels observed in Fig. 7A and B. For BTK/C1216 (Fig. 7D), the system exhibits two energy minima. One deep well around −12 kJ mol^−1^ has a narrow distribution, representing only a few conformations, while the other well at −9 kJ mol^−1^ has a broader distribution, indicating that the dominant stable states of the complex are less favorable than those of the optimal candidates, with overall moderate stability. These complexes demonstrate only moderate stability and do not show distinct advantages. Meanwhile, BTK/C5598 (Fig. 7G) this system shows a dual energy well feature. The primary well at −12 kJ mol^−1^ has a wide distribution, reflecting dominant low-energy core conformations. The secondary well at approximately −10 kJ mol^−1^ is narrower, indicating minor stable states. This pattern suggests that BTK/C5598 not only exhibits strong thermodynamic stability but also maintains certain conformational diversity, providing better binding adaptability than most candidate molecules. Together with BTK/C137, BTK/C5598 emerges as one of the most promising complexes based on the FEL analysis.

Based on these results, we ultimately selected BTK/C137 (single well reaching −13 kJ mol^−1^, highest stability) and BTK/C5598 (broad distribution at −12 kJ mol^−1^ with secondary stable states) as the top-performing candidates, demonstrating significant advantages in both stability and conformational adaptability. Our findings highlight differences in conformational stability and dynamic behavior across the simulated protein–ligand complexes, providing important theoretical insights into the relationship between conformational states and functional properties.

### 3.5. Comparable local inhibition

**Fig. 8:**
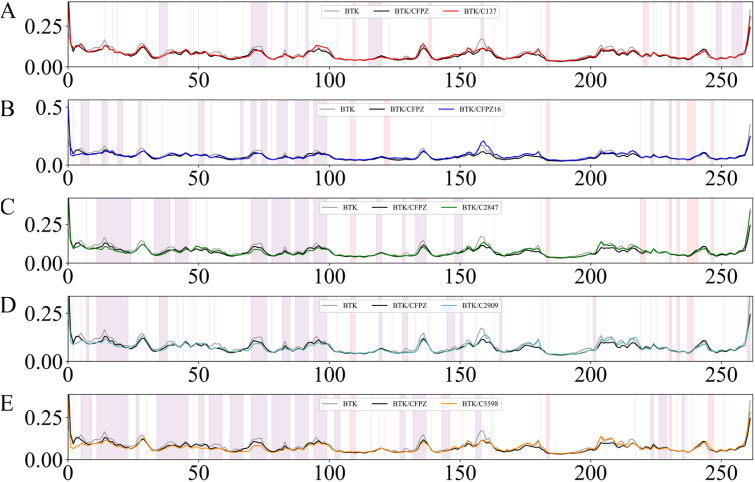
Root Mean Square Fluctuation (RMSF) values representing local fluctuations along the protein backbone. (A–E) Illustrate the RMSF profiles of BTK complexed with compounds C137, C1216, C2847, C2909, and C5598 during MD simulations. The gray line denotes the apo BTK structure, while the black line represents the BTK/CFPZ complex. The red, blue, green, cyan, and orange lines correspond to the BTK complexes with compounds C137, C1216, C2847, C2909, and C5598, respectively. Regions with a purple background indicate structural residues associated with the most effective inhibitory activity of the designed compounds, whereas regions with a red background denote residues with no observable inhibitory effect.

To further investigate the impact of the candidate compounds on the conformational stability of the BTK protein, we performed a RMSF analysis. This analysis encompassed the BTK complexes formed with compounds C137, C1216, C2847, C2909, and C5598, and was compared with both the apo BTK protein and the reference BTK/CFPZ complex. The RMSF results revealed that these candidate molecules significantly reduced the thermal motion amplitudes of the protein backbone at several key residue sites. This indicates that the compounds are capable of forming stable interactions with BTK, thereby suppressing conformational fluctuations to a certain extent, as illustrated in Fig. 8.

In Fig. 8A, the red curve (BTK/C137) shows significantly lower RMSF values within the purple-shaded region compared to both apo BTK and the reference BTK/CFPZ complex, indicating that this complex exhibits superior structural stability during the simulation. Notably, within the purple region, the RMSF values for BTK/C137 consistently remain between 0.00 and 0.15, with some intervals even dropping below 0.10. This suggests minimal structural fluctuations at key interacting residues, reflecting excellent stability. Furthermore, in the red-shaded region, the curve remains smooth without abnormal spikes, further demonstrating the system's overall stability and minimal perturbation. In contrast, in BTK/C1216 (Fig. 8B), the blue curve occasionally exceeds 0.20 within the purple region, indicating relatively lower stability. In BTK/C2847 (Fig. 8C), the green curve exhibits noticeable fluctuations, with RMSF values approaching 0.20 in some segments, suggesting poorer structural stability. Most of the values fall between those of apo BTK and the BTK/CFPZ reference complex. BTK/C2909 (Fig. 8D) shows that the cyan curve frequently exceeds 0.15 within the purple-shaded region, indicating that its stability and precision are inferior to those of Fig. 8A and E. In comparison, BTK/C5598 (Fig. 8E) shows that the orange curve maintains RMSF values consistently within the 0.00–0.10 range in the purple-shaded region, with minimal fluctuation—indicating far superior stability compared to other complexes. In the red-shaded region, the curve remains flat and stable, with no excessive rises in RMSF, further underscoring the high precision and stability of its interaction with the target residues. By integrating the RMSF analysis with data from Table 1, which outlines hydrogen bonding and hydrophobic interactions within 4 Å between compounds and BTK residues, we gain a deeper understanding of the protein–ligand interaction mechanisms. When a specific residue exhibits low RMSF values and directly participates in ligand binding, it typically reflects high structural rigidity, serving as a critical anchoring point for stable complex formation. For instance, residues such as Trp30 and Val36—if located within low RMSF regions—provide strong structural support for ligand binding through persistent hydrophobic interactions, forming the structural basis of inhibitory potency. Moreover, residues located farther from the active site, although not primary binding sites, can contribute to the inhibitory effect *via* allosteric mechanisms by modulating structural flexibility. Specifically, low-RMSF residues directly involved in interactions (*e.g.*, Trp30, Asp35) constitute the core stability zone of ligand binding, ensuring direct and effective inhibition. Meanwhile, high-RMSF interacting residues may enhance the protein–ligand microenvironment through their conformational flexibility, supporting inhibition from an allosteric perspective.

Based on the local RMSF analysis, the BTK/C137 and BTK/C5598 complexes exhibited the smallest fluctuations and highest numerical stability within the purple-shaded regions, with no significant interference observed in the red-shaded areas. Compared to other systems, these two complexes showed more pronounced and stable interactions at key binding residues, making them the best-performing groups among all analyzed systems. The RMSF analysis revealed distinct dynamic structural behaviors and inhibitory stability across the different complexes, providing valuable theoretical insights into the interaction mechanisms between small molecules and the target protein.

### 3.6. Binding free energy analysis of BTK–ligand complexes

To evaluate the binding affinity of the protein–ligand complexes, we calculated the binding free energies of the complexes using the MM-PBSA method. As shown in Table 3, the binding free energies (ΔTOTAL) for the BTK complexes with compounds C137, C1216, C2847, C2909, and C5598 are −19.24 ± 2.53 kJ mol^−1^, −15.91 ± 3.95 kJ mol^−1^, −15.08 ± 3.25 kJ mol^−1^, −13.36 ± 3.13 kJ mol^−1^, and −17.88 ± 3.73 kJ mol^−1^, respectively. In comparison, the reference complex BTK/CFPZ has a binding free energy of −8.96 ± 3.71 kJ mol^−1^. These results indicate that the generated small molecules exhibit significantly stronger binding to the BTK protein than the reference complex, demonstrating higher binding affinity. Notably, BTK/C137 and BTK/C5598 show the lowest binding free energies among all complexes, with binding abilities significantly superior to the other tested compounds (BTK/C1216, BTK/C2847, and BTK/C2909).

Further analysis of the energy components revealed that the van der Waals energies (ΔVDWAALS) for BTK/C137 and BTK/C5598 were −26.57 ± 2.70 kJ mol^−1^ and −25.07 ± 4.15 kJ mol^−1^, respectively, both significantly lower than the control complex (BTK/CFPZ: −19.7 ± 5.54 kJ mol^−1^), indicating stronger van der Waals interactions with the BTK protein. Additionally, their gas-phase binding free energies (ΔGGAS) were −33.70 ± 6.47 kJ mol^−1^ for BTK/C137 and -109.68 ± 14.35 kJ mol^−1^ for BTK/C5598, both of which are substantially lower than the control (BTK/CFPZ: −88.81 ± 16.35 kJ mol^−1^), further emphasizing their binding advantages. Although their solvation free energies (ΔGSOLV) were 14.46 ± 4.98 kJ mol^−1^ for BTK/C137 and 91.62 ± 12.78 kJ mol^−1^ for BTK/C5598, the positive values did not significantly offset the contributions of other energy components, thereby enhancing the total binding free energy. In contrast, the binding free energies of the other complexes were weaker. For example, the ΔTOTAL of BTK/C1216 was −15.91 ± 3.95 kJ mol^−1^, which, although significantly lower than the control, was still weaker than that of BTK/C137 and BTK/C5598. Its ΔVDWAALS was −23.98 ± 4.22 kJ mol^−1^, indicating a weaker non-covalent interaction with BTK. For BTK/C2847, the ΔTOTAL was −15.08 ± 3.25 kJ mol^−1^, primarily constrained by a higher solvation energy (ΔGSOLV = 18.07 ± 17.06 kJ mol^−1^), which significantly offset its binding advantage. BTK/C2909 had a ΔTOTAL of −13.36 ± 3.13 kJ mol^−1^, with its gas-phase binding free energy (ΔGGAS = −106.62 ± 22.27 kJ mol^−1^) lower than that of the control, but there is still a certain gap compared to BTK/C137.

Based on the binding free energy analysis, BTK/C137 and BTK/C5598 exhibited the best performance in terms of ΔTOTAL and key energy components (ΔVDWAALS and ΔGGAS), demonstrating significantly stronger binding affinity with the BTK protein compared to the control and other tested compounds. Through MM-PBSA analysis, we have elucidated the binding free energies and interaction mechanisms of the designed small molecules with the protein complexes, confirming the superior binding affinity of the designed molecules. This provides crucial theoretical support for subsequent drug optimization.

### 3.7. Crucial residues in kinase–inhibitor interactions by binding free energy decomposition

**Fig. 9:**
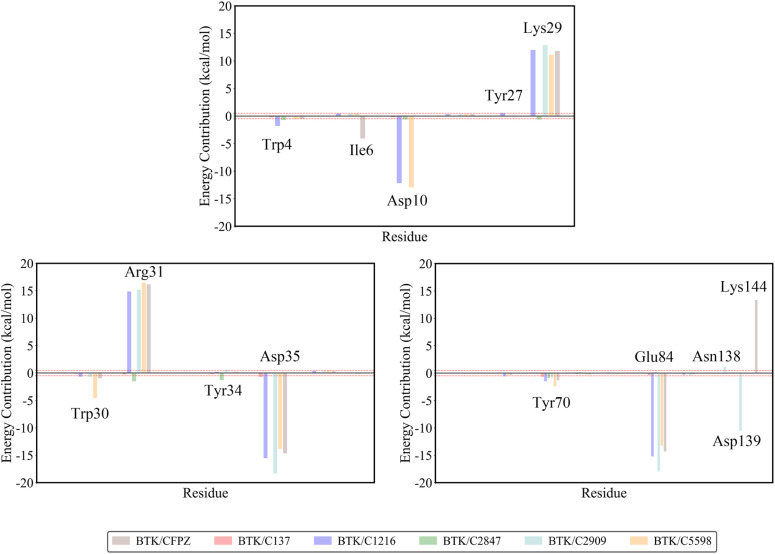
Residue decomposition contribution plot. The brown, red, blue, green, blue, and orange colors represent the residue contributions from the reference BTK/CFPZ structure, BTK complexed with compounds C137, C1216, C2847, C2909, and C5598, respectively. The *x*-axis represents the residue, and the *y*-axis represents the residue energy value. The red dashed line in the plot indicates a residue energy value with an absolute value of 0.5 kcal mol^−1^. Amino acids with an energy contribution absolute value exceeding 0.5 kcal mol^−1^ are considered key contributors to the binding.

To gain a deeper understanding of the molecular mechanisms and key interacting residues involved in the binding of candidate inhibitors to the BTK kinase, this study employs the MM-PBSA method to analyze the free energy contributions. As shown in Fig. 9, the interactions between the reference CFPZ and five candidate inhibitors (C137, C1216, C2847, C2909, and C5598) with the BTK kinase J pocket were systematically examined. The analysis focused on residues within a 6 Å radius of the inhibitor, and amino acid residues with an energy contribution absolute value greater than 0.5 kcal mol^−1^ were identified as key binding sites for the inhibitors. This approach revealed the binding specificity and optimization strategies for different inhibitors. In the reference structure BTK/CFPZ, Lys29 (11.81 kcal mol^−1^) and Arg31 (16.17 kcal mol^−1^) regulate the ligand orientation through electrostatic repulsion, while Asp35 (−14.65 kcal mol^−1^) and Glu84 (−14.29 kcal mol^−1^), as acidic residues, anchor the ligand *via* hydrogen bonds (Asp35 with the ligand hydroxyl group) and salt bridges (Glu84 with the ligand amino group), respectively. Notably, Asp35, as a core residue of the DFG motif, directly influences the inhibitor binding mode by its conformation. Furthermore, Glu84 stabilizes the catalytic center conformation through a synergistic interaction with the αC-helix.

Compared to the reference structure, the candidate inhibitors enhance their performance by precisely modulating key residue interactions. In the BTK/C137 structure, Tyr70 (−0.67 kcal mol^−1^) forms a stable π–π stacking interaction with the ligand's phenyl ring, providing greater specificity than the redundant hydrophobic interactions between Trp4 (−0.54 kcal mol^−1^) and Ile6 (−4.06 kcal mol^−1^) in the reference molecule. Meanwhile, the electrostatic contribution from Asp35 (−0.72 kcal mol^−1^) is significantly reduced (compared to −14.65 kcal mol^−1^ in the reference), maintaining the critical binding while minimizing excessive interactions with Glu84, thus reducing the off-target risk. In the BTK/C5598 structure, the indole ring of Trp30 (−4.58 kcal mol^−1^) forms a deep hydrophobic fit with the ligand's dibenzothiophene backbone, with a contribution far exceeding the combined contributions of Trp4 and Tyr70 in the reference. Additionally, the dual acidic residue cluster formed by Asp10 (−12.93 kcal mol^−1^) and Asp35 (−13.85 kcal mol^−1^) provides a more stable electrostatic complementary network than the single Asp35 in the reference, while avoiding the conformational fluctuations potentially caused by Glu84. Other structures also demonstrate unique optimization strategies, BTK/C1216 forms a hydrophobic-electrostatic complex interaction between Trp4 (−1.79 kcal mol^−1^) and Asp10 (−12.17 kcal mol^−1^) in the entrance region; BTK/C2847 reverses the electrostatic repulsion observed in the reference by forming attractive electrostatic interactions between Lys29 (−0.63 kcal mol^−1^) and Arg31 (−1.54 kcal mol^−1^); BTK/C2909 enhances stability through a dual salt-bridge network between Asp35 (−18.30 kcal mol^−1^) and Glu84 (−17.95 kcal mol^−1^) and hydrogen bonds from Asn138/Asp139.

Therefore, key residues are primarily distributed across three functional regions: the entrance regulation region (Trp4, Asp10), the charge complementarity region (Lys29, Arg31, Asp35), and the hydrophobic core region (Tyr70, Trp30). The Lys29–Arg31–Asp35 charge triad forms the core network for ligand orientation, while aromatic residues like Tyr70/Trp30 provide the main binding driving force through π–π stacking and hydrophobic effects. The candidate inhibitors overcome the binding limitations of the reference molecules by enhancing hydrophobic interactions (*e.g.*, Trp30 in BTK/C5598), reconfiguring charge networks (*e.g.*, BTK/C137 reducing excessive binding of acidic residues), and introducing novel interaction sites (*e.g.*, Asp10 in BTK/C1216). Among them, C137 achieves high selectivity through precise hydrophobic anchoring and charge balancing, while C5598 becomes the optimal candidate due to its deep hydrophobic fit and synergistic interaction with acidic residues, offering a structural paradigm for the development of high-affinity, low-toxicity BTK inhibitors.

## 4. Discussion

This study presents a novel framework for designing inhibitors targeting the J pocket of BTK kinase by integrating generative deep learning, molecular docking, and molecular dynamics simulations.^59^ We consider the BTK J-pocket to represent a prototypical cryptic pocket: its druggable state is highly dependent on the cooperative conformational regulation between the αC-helix and the DFG motif. In the absence of inhibitors, the pocket often remains “closed” or transiently open, making its formation and stable occupation extremely difficult.^60^

However, conformational sampling is far from the only barrier. First, BTK shares high structural homology with other members of the TEC family (*e.g.*, ITK, TXK) around the ATP site and the J-pocket, which drives cross-reactivity of inhibitors within the family and complicates the design of highly selective ligands.^61,62^ Second, clinical and mechanistic studies have shown that the C481S mutation in BTK or activating mutations in downstream PLCγ2 can abrogate inhibitor sensitivity, and even shifting to novel binding sites does not fully prevent resistance.^63,64^ Third, chemical design strategies are inherently a balance: while covalent inhibitors offer durable engagement, increased electrophilicity may raise off-target or toxicity risks; conversely, non-covalent inhibitors aiming for high affinity and long residence time demand precise tuning of water networks and binding kinetics.^65^ Finally, ADME and toxicological properties remain critical bottlenecks in translating “designed molecules” into “clinically viable drugs”.^66^ Therefore, we view conformational flexibility as a necessary but not sufficient challenge in designing BTK J-pocket inhibitors. Effective exploitation of this cryptic pocket will require integrated consideration of conformational sampling, selective structural engineering, resistance-mitigating strategies, chemical reactivity, water-network and kinetic optimization, as well as ADME/toxicity design. This is precisely the innovation our study aims to contribute—providing a systematic framework for developing BTK inhibitors with high selectivity, low toxicity, and strong binding affinity. Through this approach, we successfully identified high-affinity candidate molecules, C137 and C5598.

Due to the topological features of the J pocket and its critical role in allosteric regulation, traditional high-throughput screening strategies often fall short in adequately covering the conformational diversity of the allosteric site, leading to a reliance on multiple rounds of experimental validation and structural optimization—a process that is both time-consuming and inefficient.^27,67^ To address this limitation, we employed generative deep learning to gain deeper insights into the dynamic conformational characteristics of the J pocket. The generated candidates span a broader range of hydrophobic-electrostatic interaction profiles in chemical space. For instance, C5598 utilizes a dibenzothiophene scaffold to form a deep hydrophobic pocket with Trp30, while simultaneously engaging Asp35 and Asp10 to establish a dual acidic residue cluster, achieving a highly specific fit to the J pocket microenvironment. Importantly, the generative deep learning approach primarily incurs computational costs and benefits from scalable, distributed computing architectures. This enables the efficient parallel processing of massive conformational generation and screening tasks, significantly reducing reliance on physical experiments while ensuring high modeling efficiency and substantially lowering costs. Thus, it offers a cost-effective solution for large-scale conformational space exploration. To further investigate the structural diversity and representative features of the candidate molecules,^68^ we conducted clustering analysis based on molecular morphology and structural similarity. Molecules were grouped into distinct clusters (Fig. 4), and one representative compound from each cluster was randomly selected for in-depth analysis and validation. This strategy not only ensures chemical diversity among the candidates but also enhances structural novelty and target specificity in inhibitor design.

Based on the evaluation and analysis of molecular docking results (Table S1), this study demonstrates the practicality of incorporating traditional docking scores as an initial screening step. Despite limitations in modeling entropy effects and receptor flexibility, the high-throughput nature and structural interpretability of conventional docking approaches make them a valuable starting point for complex target screening. When combined with binding mode analysis, traditional methods can effectively reduce the dimensionality of candidate compounds, thereby providing high-value seed molecules for subsequent dynamic conformation optimization driven by generative deep learning models.^69^ This integrated strategy highlights the complementary strengths of computational chemistry tools at different stages of drug discovery and offers a reusable methodological framework for targeting similar biological systems.

ADMET property evaluation revealed that compounds C137 and C5598 outperformed the reference molecule CFPZ across several key parameters, including HIA, membrane permeability (BBB, MDCK), and toxicity risk (hERG inhibition, AMES mutagenicity) (Table 2). For instance, C5598 exhibited a BBB value as high as 0.979, suggesting promising potential for central nervous system drug development. Meanwhile, The AMES mutagenicity value of C137 is only 0.131, significantly lower than that of CFPZ (1.0), indicating a markedly reduced risk of carcinogenicity. These findings demonstrate that the deep learning-guided optimization strategy^70^ not only enhances binding affinity but also systematically improves drug-like properties.

Further MD simulations validated the dynamic stability of candidate small-molecule inhibitors in complex with BTK kinase.^71^ Although previous studies have employed conventional MD simulations to explore the binding pathway of fenebrutinib to the J pocket of BTK, this approach exhibits certain parameterization biases when dealing with non-covalent interactions, which can lead to deviations in the simulation results.^29^ To address these limitations, we implemented a more physiologically relevant simulation environment, including solvent conditions, pH, salt concentration, temperature, and pressure settings that closely mimic *in vivo* conditions.^72^ This refinement enhances both the biological relevance and predictive accuracy of the simulation models. Additionally, to ensure robustness and reproducibility, we conducted triplicate 100 ns all-atom MD simulations for each of the five complexes. This approach aims to overcome the limitations of traditional single-run simulations and yield more reliable binding conformations. The results demonstrated that all compounds remained stably embedded within the J pocket of BTK throughout the simulation period, with no evidence of dissociation, slippage, or significant conformational drift—indicating strong binding stability.^73^ Notably, the BTK/C137 and BTK/C5598 complexes exhibited the highest thermodynamic stability, as evidenced by minimal free energy fluctuations, low overall structural deviations, and high conformational consistency of the ligands within the binding site (Fig. 7). These findings suggest superior binding affinity and therapeutic potential for these two compounds. Further RMSF analysis (Fig. 8) revealed that key residues such as Trp30 and Val36 exhibited consistently low fluctuations throughout the simulations, indicating highly stable conformations. These residues form persistent hydrophobic interactions with the ligands, playing a crucial role in maintaining stable binding and constituting the structural foundation for inhibitory activity. Similarly, during the simulation, hydrogen bond interactions played a key role in maintaining system stability. The reference group exhibited zero hydrogen bonds, whereas the candidate complexes formed more hydrogen bonds than the control, typically 1–2 additional bonds, while non-covalent interactions were comparatively weaker (Fig. S3). Taken together, this study underscores the value of integrating molecular docking with molecular dynamics simulations.^74^ Such an approach effectively compensates for the limitations of static structural assessments, enhances mechanistic understanding of drug–target interactions, and provides critical insights for the selection and optimization of lead compounds.^75^ This integrative strategy demonstrates strong application potential and practical value in structure-based design of BTK inhibitors.

From a mechanistic perspective, van der Waals and electrostatic interactions between molecules are key driving forces in maintaining the stable binding of small-molecule ligands to their target proteins.^76^ To quantitatively evaluate the binding affinity of candidate inhibitors to the BTK protein, binding free energies were calculated based on MD simulations at the equilibrium stage and representative structural ensembles (Table 3). The results showed that all candidate compounds exhibited more favorable binding free energies compared to the reference molecule. Among them, the BTK/C137 and BTK/C5598 complexes demonstrated the lowest total binding free energies and the most favorable van der Waals energy contributions, indicating the formation of more stable and higher-affinity binding modes with BTK kinase. Further energy decomposition analysis revealed that van der Waals interactions were the primary contributors to high binding affinity,^77^ far outweighing electrostatic or polar solvation components. This highlights the dominant role of hydrophobic interactions in the recognition of BTK inhibitors (Table 3). Key residue decomposition analysis uncovered the core interaction network of J pocket inhibitors, Lys29 and Arg31 act as directional anchoring points through electrostatic complementarity, while Trp30 and Tyr70 enhance binding stability *via* π–π stacking and hydrophobic effects. Notably, C137 significantly reduced its reliance on Asp35 (with electrostatic contribution decreasing from −14.65 kcal mol^−1^ in CFPZ to −0.72 kcal mol^−1^), suggesting a refined binding mechanism. Further analysis of the structure–energy relationships across different candidates revealed that C137 and C5598 achieved optimized binding through distinct mechanisms, markedly enhancing their binding selectivity.^78,79^ These findings provide a promising structural paradigm for the future development of BTK inhibitors with improved affinity, selectivity, and reduced toxicity.^80^

**Table 3:** Calculated binding free energies of protein-small molecule complexes^a^

Energy component	BTK/CFPZ (kJ mol^−1^)	BTK/C137 (kJ mol^−1^)	BTK/C1216 (kJ mol^−1^)	BTK/C2847 (kJ mol^−1^)	BTK/C2909 (kJ mol^−1^)	BTK/C5598 (kJ mol^−1^)
ΔVDWAALS	−19.7 ± 5.54	−26.57 ± 2.70	−23.98 ± 4.22	−24.95 ± 4.28	−20.36 ± 3.60	−25.07 ± 4.15
ΔEEL	−70.11 ± 14.22	−7.14 ± 5.18	−86.04 ± 19.42	−8.20 ± 17.94	−86.26 ± 22.43	−84.46 ± 12.81
ΔGGAS	−88.81 ± 16.35	−33.70 ± 6.47	−110.02 ± 20.7	−33.14 ± 18.16	−106.62 ± 22.27	−109.68 ± 14.35
ΔGSOLV	80.85 ± 14.92	14.46 ± 4.98	94.11 ± 18.93	18.07 ± 17.06	93.26 ± 22.16	91.62 ± 12.78
ΔTOTAL	−8.96 ± 3.71	−19.24 ± 2.53	−15.91 ± 3.95	−15.08 ± 3.25	−13.36 ± 3.13	−17.88 ± 3.73

a ΔVDWAALS = van der Waals energy; ΔEEL = electrostatic energy; ΔGGAS = gas-phase binding free energy; ΔGSOLV = solvation free energy; ΔTOTAL = estimated binding free energy.

However, our study still has certain limitations. Although molecular dynamics simulations demonstrated that C137 and C5598 maintained stable conformations over 100 ns, their dynamic behavior on longer timescales (*e.g.*, in the microsecond range) and their *in vivo* pharmacokinetic profiles remain to be experimentally validated. Moreover, the chemical space explored by the generative model is inherently constrained by existing structural data, which may overlook rare or unresolved conformations within the J pocket. Future work could integrate cryo-EM-derived dynamic conformational data to further refine and enhance the model.

## 5. Conclusion

In this study, we proposed a computational framework that integrates generative deep learning, molecular docking, and MD simulations to explore pocket-aware inhibitors targeting the BTK kinase J pocket. Through a multi-step computational screening process involving molecular clustering, docking, and drug-likeness evaluation, we successfully identified 5 candidate molecules from a library of 10 000 pocket-aware designed compounds. Further MD simulations comparing these candidates with the reference inhibitor CFPZ revealed that two compounds, C137 and C5598, exhibited superior conformational stability, inhibitory performance, and binding free energy. Subsequent energy decomposition analysis highlighted a synergistic mechanism of hydrophobic interactions and electrostatic networks that underpins the high binding efficiency of these compounds. Compared with traditional design strategies, our approach overcomes the limitations of static structural models by dynamically capturing the conformational diversity of the J pocket, achieving comprehensive optimization in affinity, selectivity, and drug-like properties of the inhibitors. This lays a solid foundation for subsequent preclinical development. Future research may combine experimental validation with iterative model refinement to further explore the therapeutic potential of J pocket inhibitors in B-cell malignancies and autoimmune diseases, promoting the translational application of dynamic conformation-based precision drug design.

## Author contributions

Li-Ting Zheng: data curation, methodology, writing-original draft. Kun Qian: formal analysis. Jun Zhang: methodology. Meng-Ting Liu: methodology. Yi Li: conceptualization, methodology, writing-original draft, writing-review & editing. Li-Quan Yang: conceptualization, supervision, writing-review & editing.

## Conflicts of interest

The authors declare that they have no competing interests.

## Supplementary Material

RA-015-D5RA04840K-s001

## Data Availability

The data we can make openly available is confirmed to be at this link: https://github.com/liyigerry/kinase. Supplementary information: screening and validation data (affinity scores, ADMET); molecular dynamics simulation-related data (RMSD, Rg, changes in hydrogen bonds and non-covalent interactions). See DOI: https://doi.org/10.1039/d5ra04840k.
