# Supplementary material for: Exploring pocket-aware inhibitors of BTK kinase by generative deep learning, molecular docking, and molecular dynamics simulations
Source: RSC Adv. 2025 Sep 25;15(42):35336–55. doi: 10.1039/d5ra04840k (PMC12461837; doi:10.1039/d5ra04840k)
Supplement: RA-015-D5RA04840K-s001 [file RA-015-D5RA04840K-s001.pdf]

Supplementary Information (SI) for RSC Advances.
This journal is © The Royal Society of Chemistry 2025

Exploring pocket-aware inhibitors of BTK kinase by generative deep learning, molecular docking, and molecular dynamics simulations

Li-Ting Zheng[abc], Kun Qian[d], Jun Zhang[e], Meng-Ting Liu[abc], Yi Li[bce]*, Li-Quan Yang[abc]*

[a] College of Agriculture and Biological Science, Dali University, Dali, China.

[b] Key Laboratory of Bioinformatics and Computational Biology, Department of Education of Yunnan Province, Dali University, Dali, China.

[c] Co-Innovation Center for Cangshan Mountain and Erhai Lake Integrated Protection and Green Development of Yunnan Province, Dali University, Dali, China

[d] College of Basic Medicine, Dali University, Dali, China.

[e] College of Mathematics and Computer Science, Dali University, Dali, China.

* Corresponding authors: ylqbioinfo@gmail.com.(Li-Quan Yang), yili@dali.edu.cn.(Yi Li)

Table S1. Affinity scores and clusters of 25 candidate compounds and reference compound CFPZ with BTK protein

Complex	Affinity score (kcal mol^{-1})	Cluster number
BTK/CFPZ	-6.8	/
BTK/C 4492	-6.7	1
BTK/C 6406	-6.2	2
BTK/C 2909	-7.4	3
BTK/C 8760	-6.6	4
BTK/C 7592	-7.2	5

Complex	Affinity score (kcal mol^{-1})	Cluster number
BTK/C 1399	-7.3	6
BTK/C 3619	-6.8	7
BTK/C 387	-6.5	8
BTK/C 2847	-8.5	9
BTK/C 3715	-6.9	10
BTK/C 3070	-6.8	11
BTK/C 1542	-6.4	12
BTK/C 1216	-7.3	13
BTK/C 5598	-7.4	14
BTK/C 6346	-6.3	15
BTK/C 6902	-8.2	16
BTK/C 1609	-6.7	17
BTK/C 9539	-7.1	18
BTK/C 9122	-6.7	19
BTK/C 137	-7.0	20
BTK/C 4286	-6.5	21
BTK/C 9026	-8.6	22

Complex	Affinity score (kcal mol^{-1})	Cluster number
BTK/C 4326	-6.6	23
BTK/C 7188	-6.8	24
BTK/C 9844	-6.0	25

Table S2. The ADMET(Absorption, Distribution, Metabolism, Excretion, and Toxicity) spectra of 10 candidate compounds and the reference compound CFPZ.

Compound	Caco-2	HIA	BBB	MDCK	Pgp inhibitor	Pgp substrate	hERG Blockers	AMEs Muta genicity	Carcinogenicity	Acute Toxicity Rule	H-HT
CFPZ	-4.965	0.0	0.313	-4.706	0.139	0.999	0.0	0.0	1.0	0	0.999
C137	-4.866	0.004	0.01	-4.479	0.002	0.007	0.131	0.09	0.11	0	0.664
C2847	-4.687	0.004	0.014	-4.715	0.0	0.027	0.182	0.263	0.33	0	0.68
C5598	-4.641	0.0	0.979	-4.829	0.78	0.16	0.434	0.235	0.297	0	0.698

Compound	Caco-2	HIA	BBB	MDCK	Pgp inhibitor	Pgp substrate	hERG Blockers	AMEs Muta genicity	Carcinogenicity	Acute Toxicity Rule	H-HT
C2909	-4.963	0.137	0.486	-4.625	0.995	0.263	0.771	0.286	0.051	0	0.442
C1216	-5.048	0.002	0.939	-5.072	0.869	0.025	0.577	0.565	0.122	0	0.551
C7592	-4.448	0.006	0.078	-4.673	0.908	0.442	0.85	0.943	0.719	0	0.591
C1399	-4.543	0.0	1.0	-4.726	1.0	0.037	0.537	0.221	0.242	0	0.787
C6902	-4.574	0.003	0.033	-4.557	0.424	0.046	0.685	0.646	0.832	0	0.746
C9539	-4.7	0.0	0.798	-4.815	0.453	0.025	0.149	0.892	0.946	0	0.781
C9026	-4.824	0.0	0.4	-4.618	0.671	0.6	0.411	0.284	0.237	0	0.739

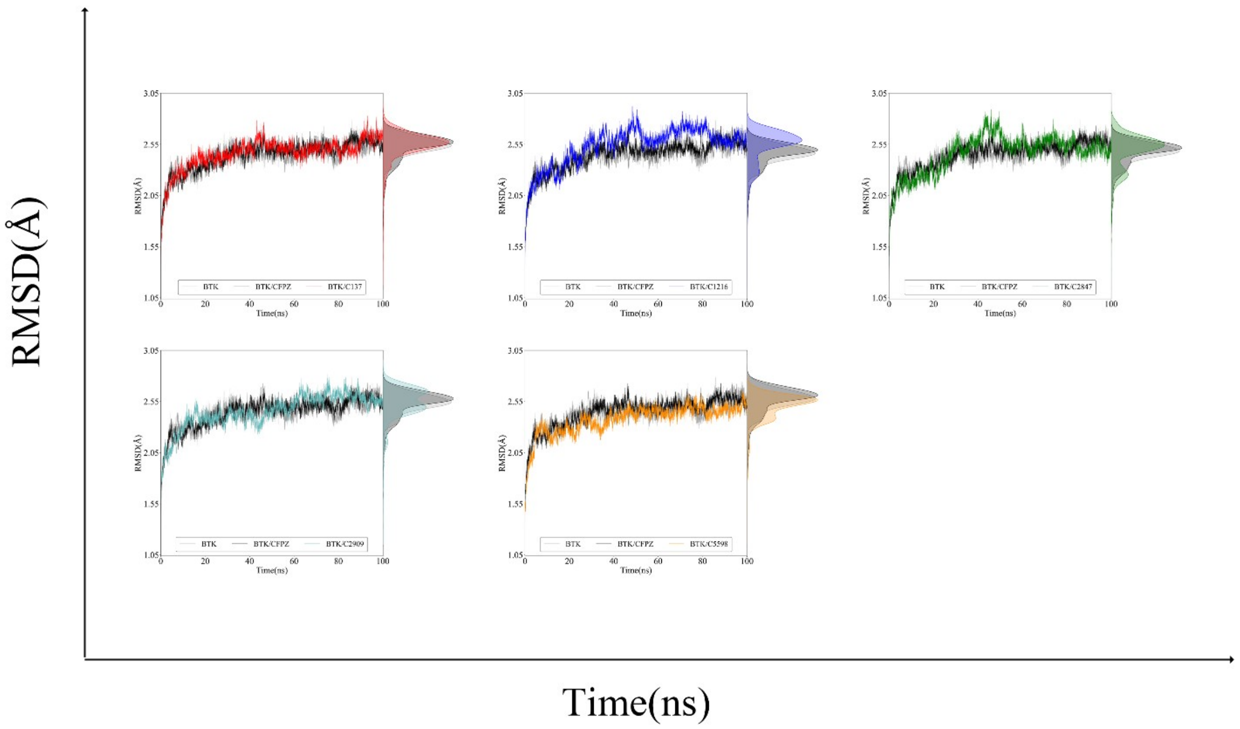

Figures S1. Root Mean Square Deviation (RMSD) plots of the protein backbone over 100 ns for five protein–ligand complexes (including apo BTK and BTK/CFPZ). The gray line represents the RMSD of apo BTK (the ligand-free form of BTK), the black line represents BTK/CFPZ (BTK in complex with the ligand CFPZ), and the red, blue, green, cyan, and orange lines represent BTK/C137, BTK/C1216, BTK/C2847, BTK/C2909, and BTK/C5598, respectively.

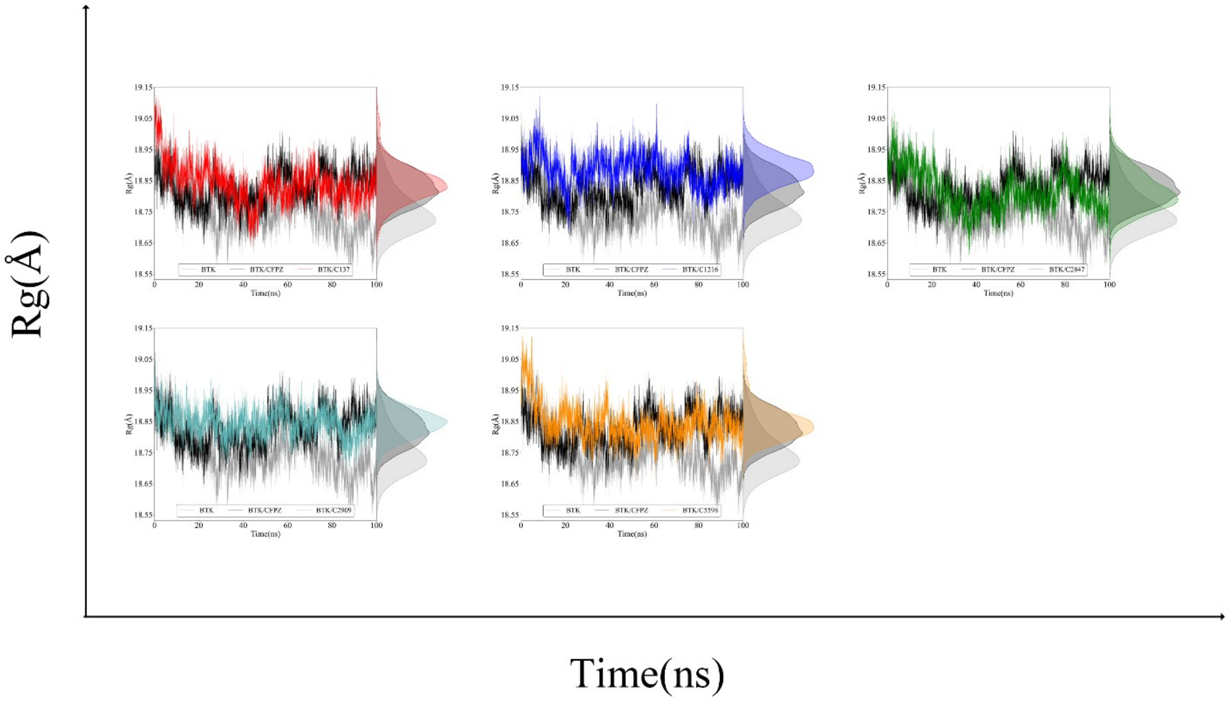

Figures S2. Radius of Gyration (Rg) plots of the protein backbone over 100 ns for five protein–ligand complexes (including apo BTK and BTK/CFPZ). The gray line represents the Rg of apo BTK (the ligand-free form of BTK), the black line represents the complex BTK/CFPZ, and the red, blue, green, cyan, and orange lines represent BTK/C137, BTK/C1216, BTK/C2847, BTK/C2909, and BTK/C5598, respectively.

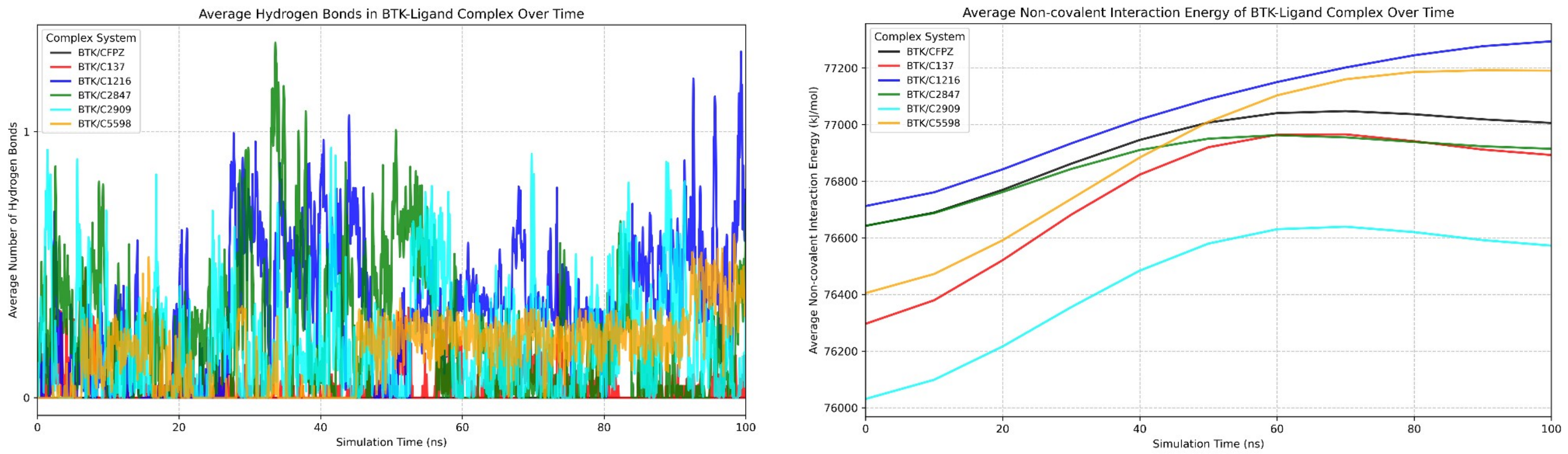

Figures S3. Interaction profiles of six protein–ligand complexes over 100 ns. The black line represents the BTK/CFPZ complex, while the red, blue, green, cyan, and orange lines correspond to BTK/C137, BTK/C1216, BTK/C2847, BTK/C2909, and BTK/C5598, respectively. The left panel shows the variation in hydrogen bond interactions, and the right panel illustrates the changes in non-covalent interactions.
